# Preparation and Investigation of Sustained-Release Nanocapsules Containing Cumin Essential Oil for Their Bacteriostatic Properties

**DOI:** 10.3390/foods13060947

**Published:** 2024-03-20

**Authors:** Mingcheng Zhang, Mingyang Li, Danyang Zhang, Ying Yu, Kaixian Zhu, Xiaodan Zang, Dengyong Liu

**Affiliations:** 1 College of Food Science and Technology, Bohai University, Jinzhou 121013, China; 2 Cuisine Science Key Laboratory of Sichuan Province, Sichuan Tourism University, Chengdu 610100, China; 3 College of Public Health, Food Quality and Safety, Mudanjiang Medical University, Mudanjiang 157011, China

**Keywords:** cumin essential oil, nanocapsules, chitosan, antibacterial activity

## Abstract

Cumin essential oil chitosan nanocapsules (CENPs) were prepared through the ionic gelation method by blending chitosan (CS) with cumin essential oil (CEO) in different proportions (1:0.8, 1:1, 1:2, 1:3, 1:4). Subsequently, these nanocapsules were characterized and evaluated for their antibacterial properties to determine the optimal cumin essential oil encapsulation and antibacterial efficacy. The outcomes demonstrated that the highest encapsulation efficiency of CENPs was 52%, achieved with a 1:3 CS/CEO ratio. At this point, the nanoparticles had the smallest particle size (584.67 nm) and a regular spherical distribution in the emulsion. Moreover, the CENPs could release the encapsulated CEOs slowly, leading to efficient inhibition of *E. coli* and *L. monocytogenes* over a relatively extended period (24–36 h) compared to the CS and CEO. This research offers a promising approach for the use of nanocapsules in food preservation.

## 1. Introduction

Essential oils, as an additive for natural foods, are oily, aromatic, and volatile extracts derived from a variety of plant sources. They possess a broad range of antimicrobial properties, making them widely used in pharmaceuticals, cosmetics, and food industries [1,2]. Their natural preservative properties make them commonly used as antioxidants to scavenge for free radicals and chelate metals [3]. For instance, research indicates that black pepper essential oil is capable of preventing the contamination of pork tenderloin with microorganisms such as *Pseudomonas* spp. and *Enterobacteriaceae*. Additionally, it can help reduce lipid oxidation [4]. Similarly, ginger essential oil can extend the shelf life of chicken breasts by decreasing the total amount of aerobic chilling bacteria, molds, and yeasts present [5]. The combination of cumin essential oil and low-dose gamma irradiation was found to effectively control the microflora and inoculum of pathogenic bacteria, thus safeguarding the microbiological safety of beef during prolonged refrigeration [6].

Cumin (*Cuminum cyminum* L.) belongs to the Umbelliferae family and is widely used as a flavoring agent due to its unique taste, making it the second most popular spice worldwide [7]. Cumin essential oil (CEO), extracted from the seeds, contains volatile compounds such as cumin aldehyde, γ-pinene, and β-pinene [8]. Several studies indicate that cumin essential oil (CEO) has potent antioxidant and antimicrobial properties against bacteria, including *S. aureus*, *E. coli*, *L. monocytogenes*, and *S. typhimurium* [9,10]. However, CEO’s high volatility, low solubility, and instability can lead to the functional failure of small food doses when added directly. When used in large quantities, the potent and overpowering taste of cumin essential oils can have a negative impact on the flavor of food products. This impedes the use of cumin essential oils in food preparation [11,12].

Nanocapsule technology is an innovative approach that utilizes nanocomposites, nanoemulsification, and nanoconstruction techniques to encapsulate substances in nanoscale capsules and produce microcapsules. This technology provides a larger surface area, which in turn increases the efficiency of essential oil encapsulation [13,14]. At the same time, nanocapsules can enhance the functionality of essential oils by providing high stability and controlled release properties. This can lead to improved antioxidant, antimicrobial, and sensory properties [15,16]. Chitosan nanocapsules containing essential oils of clove were created by Xu et al. employing ionic gel technology. The nanocapsules exhibited prolonged and potent antimicrobial activity against pathogenic bacteria isolated from fresh blueberries [17]. Zhang et al. utilized the ionic gelation technique to develop chitosan gelatin coatings infused with tarragon essential oils (TEOs). Their findings revealed that nano-encapsulation enabled a gradual release of TEOs, leading to enhanced antioxidant, antimicrobial, and sensory characteristics [18]. Chitosan is a packaging material highly valued for nanoparticle preparation due to its excellent biocompatibility, degradability, and inherent antimicrobial, emulsification, and film-forming properties [19,20]. When dissolved under acidic conditions, the -NH_2_ group of chitosan gets actionized to form -NH^3+^. This capability allows it to interact with negatively charged polymers and contribute to the formation of nanocapsules [21].

The aim of this study was to prepare chitosan nanocapsules (CENPs) containing cumin essential oil using the ionic gelation method and to characterize the CENPs. The antibacterial activity of the CENPs against *E. coli* and *L. monocytogenes* was investigated by measuring the slow release of essential oils. Additionally, the effect of CENPs on the preservation of stored mutton was evaluated to propose a new method for preserving mutton.

## 2. Materials and Methods

### 2.1. Materials

Cumin essential oil (CEO NF504) was sourced from Zhongjing Food Co., Ltd. (Henan, Nanyang, China). Chitosan (CS, MW 280 kDa, degree of deacetylation > 90%) was purchased from Beijing Solarbio Science & Technology Co., Ltd. (Beijing, China). Other chemicals were provided by Sinoharm Chemical Reagent Co., Ltd. (Shanghai, China) and were of at least analytical grade. The hindquarter meat from 12-month-old Sunit sheep were purchased from Yang-yang Animal Husbandry Co., Ltd. (Inner Mongolia Autonomous Region, China). *Escherichia coli* (ATCC 25922) and *Listeria monocytogenes* (ATCC 33090) were isolated in our laboratory.

### 2.2. Synthesis of CENPs

Cumin essential oil chitosan nanocapsules (CENPs) were synthesized and optimized following an established method. Chitosan was completely dissolved in a 1% (*v*/*v*) aqueous acetic acid solution, resulting in the formation of chitosan/acetic acid solution (CAA) (3.2 mg/mL) via stirring in a water bath at 60 °C for 2 h. The pH value of the CAA solution was adjusted to 4.8 using a 2M NaOH solution. Finally, Tween 80 was added to the solution according to the ratio of CEO to Tween 80 as 1:0.8 (*m*/*m*). The homogenous emulsion was obtained through magnetic stirring at 60 °C for 1 h. Various amounts of CEO with distinct CS/CEO ratios—1:0.8, 1:1, 1:2, 1:3, and 1:4—was introduced to the homogenous emulsion. It was stirred under magnetic force for another hour to form a CEO/CS oil-in-water emulsion at room temperature. Subsequently, a Sodium Tripolyphosphate solution (TPP) was included in the emulsion at a CS-to-TPP ratio of 3:1 (*m*/*m*). It underwent 2 h of stirring to crosslink. The sediment was collected via washing at 9000 rpm for 20 min, followed by dispersion in deionized water. Lyophilization was conducted for 48 h, and the resulting product was stored at 4 °C for subsequent analysis.

### 2.3. Characterization of CENPs

#### 2.3.1. Particle Size and Zeta Potential

The CENP samples were prepared by dispersing in distilled water and detected by a Malvern Zetasizer Nano ZS ZEN3600 analyzer (Malvern Instruments Ltd., Malvern, UK).

#### 2.3.2. Embedding Efficiency (EE)

The concentration of CEO embedded within CENPs was determined using a UV–Visible Spectrophotometer (UV-2550, Shimadzu, Kyoto, Japan) following the method developed by Lalita Keawchaoon [22]. Briefly, a 200 μL sample with a concentration of 10 mg/mL was mixed with 5 mL of 2M HCl and kept in a boiling water bath for 30 min. Subsequently, 2 mL of ethanol was added, and the mixture was centrifuged at 7000 rpm for 5 min at room temperature. The absorbance of the supernatant was measured at wavelengths between 250 and 400 nm. The highest absorption was recorded at a wavelength of 310 nm and the quantity was subsequently calculated using the standard curve generated from various concentrations of CEO in absolute ethanol at 310 nm. Three measurements were taken. The embedding efficiency (EE) of the CEO/CENP ratio was computed using (1), respectively.

(1) $$EE\left(\%\right)=\frac{Weight\;of\;embed\;CEO}{Weight\;of\;initial\;CEO}\;$$

#### 2.3.3. Scanning Electron Microscopy (SEM)

The lyophilized samples (0.5 × 0.5 cm) were fixed on the copper plate then observed after spraying with gold by scanning electron microscopy (S4800, Hitachi, Japan) with an accelerating voltage of 5.00 kV.

#### 2.3.4. Microscopy

The microscopic nanocapsule images were observed with a vertical microscope (Nikon 80i, Tokyo, Japan) at room temperature. Nanocapsules (5 μL) were dropped on the glass slide, then a coverslip was placed onto the glass slide to remove air in nanocapsule. The images were collected using elementary imaging analysis software (NIS-Elements, Nikon, Melville, NY, USA).

#### 2.3.5. Confocal Laser Scanning Microscopy (CLSM)

The staining method for CENPs was performed according to the method of Huang with slight modification [23]. The CS was first dissolved in acetic acid (200 mL, 0.1 mol/L), and a 10 mg/mL CS solution was obtained. FITC-labeled CS was synthesized by adding 100 mL of dehydrated methanol followed by 10 mL of FITC in methanol (2.0 mg/mL) to CF solution in the dark under the heating condition of 70 °C water bath. After dying for 24 h under a 150 rpm magnetic stirrer, the CS solution was stained (15,000 Da) for three days under dark conditions and then lyophilized for 48 h to obtain FITC-labeled CS. Nanocapsules were prepared from FITC-labeled CS and cumin essential oil with Nile red staining solution. The samples were diluted before placing on a slide glass with a coverslip.

CLSM (Leica Microsystems, Inc., Heidelberg, Germany) was employed to image the distribution of CENPs in the above sections. FITC and Nile red were excited with a laser at wavelengths of 495 nm and 625 nm, respectively. Image acquisition was performed using a 60× oil lens. Two-dimensional image layers of 512 × 512 pixel size with 12 bits per pixel were acquired and saved in the CLSM image format.

#### 2.3.6. Determination of CEO Release from CENPs

The release of CEO was determined according to the method of Hosseini [21]. Freeze-dried CEO-loaded chitosan nanoparticles (20 mg) were placed in a bag filter (2000 Da) containing 5 mL of 60% phosphate-buffered saline (PBS, pH 7.4). Then, the bag filter was put into a sealed beaker containing 50 mL phosphate buffer and shaken at a constant temperature of 4 °C. After a certain time, solution of 4.0 mL was taken out at intervals, and the same volume of new PBS was added in. The absorbance of CENPs was measured at 310 nm by UV–Visible Spectrophotometer (UV-2550 Shimadzu, Japan), and the release curves were drawn.

#### 2.3.7. Differential Scanning Calorimetry (DSC)

The thermal stability of nanocapsules was determined using Discovery series DSC (Q2000, TA instruments, New Castle, USA). According to the test method described by Cansu with slight modifications [24], 5–6 mg of CS powder, CEO, and lyophilized CENPs powder were selected for determination and analysis. The test temperature range was 30–450 °C, and the heating rate was 20 °C/min, nitrogen rate was 100 mL/min.

#### 2.3.8. Antibacterial Activity

The agar well diffusion method was performed to verify the antibacterial activity using some modifications. To select strains, we referred to and modified the method of Hasani-Javanmardi et al. Ultimately, we chose *E. coli* and *L. monocytogenes* as indicator strains to assess the antimicrobial activity of CENPs [10]. Firstly, sterilized Oxford cups were put on a sterile dish, and 10 mL of nutrient agar containing 1.0 × 10^7^ CFU/mL of indicator strain was slowly added. After the agar solidified, the Oxford cups were removed and 100 μL of 1 mg/mL CENPs was added to the resulting hole on the dish. Petri dishes were then incubated at 37 °C for another 24 h and the inhibition zones were observed.

### 2.4. Application of Nanocapsules on Mutton Preservation

The hindquarter meat from 12-month-old Sunit sheep were purchased from Yang-yang Animal Husbandry Co., Ltd. (Inner Mongolia Autonomous Region, China) and transported to the laboratory through the cold chain (−18 °C). Then, the mutton was cut into 10 × 10 × 10 mm^3^ cubes and randomly divided into three groups. With the aid of the micropipette, 5 mL of pure CEO, CS solution, and CENPs prepared in the preceding paragraph was measured and distributed evenly over the surface of the mutton, wrapped in cling film and stored in the fridge. A total of four sets of samples were used for later examination: Control, CS, CEO, CENPs.

#### Microbiological Analyses

Samples were obtained on days 0, 1, 3, 5, and 7 during the refrigeration of mutton using four sets of mutton samples. From each set, 10 g of meat samples were weighed, mixed with 90 g of 0.1% peptone water, and homogenized for 1 min at 200 rpm in a Stomacher. Then, series of dilutions (up to 10^−9^) were prepared using the same peptone water, and inoculated into Petri dishes, which were previously mixed with medium, for determination purposes. Total counts of viable bacteria and cryophilic bacteria were determined in accordance with National Standard of the People’s Republic of China GB 4789.2-2022 during cold storage [25].

### 2.5. Statistical Analysis

All specific experiments were carried out in triplicate. Three independent experimental trials (replications) were conducted, and the results are displayed as the mean values ± standard deviations. Statistical analysis was processed by the statistical software SPSS 20.0 (SPSS, Inc., Chicago, IL, USA). The significant analysis of variance of test groups was carried out using the Duncan test, and differences were considered significant at *p* < 0.05.

## 3. Results and Discussion

### 3.1. Encapsulation Efficiency (EE) of CENPs

Encapsulation efficiency was widely used to characterize the percentage coverage of encapsulated CEO in CENPs. A UV–Visible Spectrophotometer analysis at a wavelength of 310 nm was performed to measure the CEO content in the CENPs, and the results are shown in Table 1. At a mass ratio of 1:0.8 (*m*/*m*) of CS to CEO, the efficiency of CENPs was 38.13%. With an increase in the mass ratio of CS to CEO, the efficiency of CENPs slowly rose and reached its peak value of 52.00% at a mass ratio of 1:3 (*m*/*m*) of CS to CEO. With a further increase in the amount of CEO, the efficiency of CENPs decreased to 37.68%. At low CS content in the system, free cumin essential oil adheres to the surface of CENPs, leading to their aggregation and decreasing EE [26]. More free oil embedded in the wall material gradually increases the EE, but further embedding of a large amount of oil saturates the wall material and decreases the EE of CEO nanocapsules, a phenomenon consistent with that observed in studies [27].

### 3.2. Characterization of CENPs

#### 3.2.1. Particle Size and Zeta Potential

The particle size, zeta potential, and polydispersity index (PDI) of CENPs are presented in Table 1. As depicted in Table 1, CENPs with varying sizes (584.67–810 nm) were synthesized by the ion–gel method by adjusting the ratio of chitosan (CS) to cumin essential oil (CEO). At a CS/CEO ratio of 1:0.8, the average particle size of CENPs was found to be maximum, measuring 810.00 nm. This increment in particle size can be primarily attributed to the higher concentration of chitosan [28]. Furthermore, the presence of excess emulsifiers due to the addition of smaller amounts of essential oil resulted in agglomeration, further contributing to the increase in particle size. As more CEO was added, the particle size progressively decreased, reaching a minimum of 584.67 nm at a CS/CEO ratio of 1:3. This ratio signified that the CEO was being gradually encapsulated, a finding also corroborated by the encapsulation efficiency (EE). However, as more CEO was added, the particle size escalated again and reached 618.67 nm. This was attributed to a decline in EE and the aggregation of excessive CEO, which led to the formation of larger droplets.

The zeta potential governs the degree of electrostatic attraction or repulsion between particles and is instrumental in various phenomena, such as aggregation or dispersion. This parameter plays a crucial role in assessing the stability of suspensions, emulsions, and dispersions [29]. It is widely acknowledged that nanoemulsions with zeta potential values exceeding 30 mV or below −30 mV is generally considered to be physically stable suspensions [24]. Increased zeta potential values of nanoemulsions indicate heightened electrostatic repulsion between dispersed droplets, which, in turn, signifies superior stability of dispersed nanoparticles [30]. In this study, CENPs were found to remain stable even with various CEO additions due to their high positive zeta potential values, attributed to the protonation of chitosan amino groups. At a ratio of CS to CEO of 1:0.8, the zeta potential was +29.03 mV, and this charge marginally decreased to +25.80 mV with an increase in CEO addition. This phenomenon is attributed to the ARMOR effect of the positively charged CEO. As the ratio of CEO increased to a CS/CEO ratio of 1:3, the zeta potential reached its maximum value of +33.77 mV, indicating the optimal stability of the prepared CENPs. With further CEO addition, the zeta potential value decreased to +27.13 mV due to excessive coalescence of uncoated CEO droplets. This resulted in larger particle sizes, ultimately leading to a decrease in repelling forces between the particles [26]. Zeta potential serves as an essential parameter in studying particle characteristics and contributes to determining their stability and antibacterial potential [31].

Polydispersity index (PDI) is a measure of the dispersion of polymers. A lower PDI value signifies a higher degree of sample homogeneity, emphasizing the uniformity of particle diameter [32]. Our findings reveal a PDI value of 0.276 for a CS/CEO ratio of 1:0.8. As the CS/CEO ratio increases, the PDI value correspondingly escalates, reaching a maximum of 0.354 at a ratio of 1:3. However, it subsequently decreases to 0.143 with an increasing number of CEOs. These results suggest that the optimal dispersion of CENP occurs at a CS/CEO ratio of 1:3.

#### 3.2.2. Morphology of CENPs

**Figure 1:**
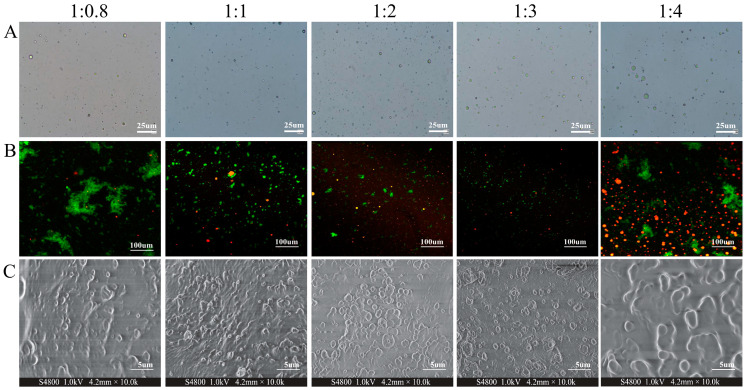
Microscopy images (**A**), CLSM images (**B**), and SEM images (**C**) of CENPs with different chitosan and cumin essential oil ratios (1:0.8, 1:1, 1:2, 1:3, 1:4).

The morphological structure of nanocapsules has a significant impact on their properties. The retention capacity and functionality of microcapsules rely heavily on the integrity of their shell material. Moreover, the mobility of nanocapsules is also contingent upon their particle morphology [33]. Figure 1A displays the morphology of the nanocapsules produced in this experiment, which exhibited a symmetrical, homogeneous aggregation with a regular spherical structure. As CEO is gradually added, the number of observable nanoparticles also increases until it reaches its maximum at a CS/CEO ratio of 1:3 in line with the encapsulation efficiency outcomes reported in the previous research.

Conventionally, CSLM is employed to observe nanoparticle morphology, and Figure 1B illustrates the morphology of nanoparticles containing cumin essential oil and chitosan encapsulation under laser confocal microscopy. Where the red represents cumin essential oil stained by Nile Red stain and green represents chitosan stained with FTIC, it is evident from the figure that the overall color at the CS/CEO ratio of 1:0.8 is green with a few sporadic red spots. At present, the low addition of essential oils means that numerous chitosan did not get a chance to encapsulate them. As a result, chitosan is present as monomers or in aggregates, forming large chitosan clusters that appear as big green clusters in the illustration. Therefore, the opportunity to encapsulate the essential oils was limited. When the ratio of chitosan (CS) to cumin essential oil (CEO) reached 1:1, the previously present large clusters of green particles gradually vanished. They were subsequently supplanted by numerous evenly dispersed green spherical particles containing a few interspersed red patches. The red patches are believed to indicate the unembedded CEOs. As the ratio of chitosan to essential oil (CS/CEO) approached 1:3, the size of the green spherical particles reduced notably under increasing concentrations of essential oils. Close observations of the corresponding images revealed densely packed green particles, with the color of the particles being noticeably darker than other images. This may arise from the incorporation of red CEOs, resulting in the encapsulation of most chitosan particles and consequent color changes. Notably, as the ratio of essential oil addition (CS/CEO = 1:4) continued to increase and surpassed the encapsulation capacity of chitosan, a significant quantity of CEOs was dispersed in the image resulting in numerous red spots being displayed in the picture. This aligns with the findings from the optical microscopy results (Figure 1A) and the encapsulation efficacy (Table 1).

The SEM was used to evaluate the external morphology of the CENPs as presented in Figure 1C. These data are valuable in determining particle size and morphology. The analysis of dried particles provides crucial insight into the particles, and the chemical and physical factors that affect their structure [34]. The figure demonstrates that CENPs possess a predominantly spherical shape, which is consistent with findings by Wang et al. in their investigation of EOs@PDA nanoparticles, wherein the predominant morphology was spherical [35]. However, it is noteworthy that the particle sizes of the synthesized CENPs differed from those of other studies, potentially due to differences in the synthesis methods and embedding materials used [36]. For instance, the microcapsules that incorporated gum arabic exhibit typical monoclinic crystalline structures and possess a particle size of 8.20 mm [37]. After introducing CS/CEO in a 1:0.8 ratio, the number of produced nanocapsules was limited and their distribution was sparse. However, as the amount of CEO increased gradually, more nanocapsules were prepared. Ultimately, when the ratio of CS/CEO was 1:3, the formation of spherical nanocapsules was most prevalent and uniform throughout. The continuous addition of essential oils resulted in oil aggregation and the formation of large clusters. This occurrence appears to be linked to the SEM sample preparation’s drying process, where surplus essential oils adhered to the nanocapsules, leading to partial essential oil clustering [17].

#### 3.2.3. Thermal Stability

**Figure 2:**
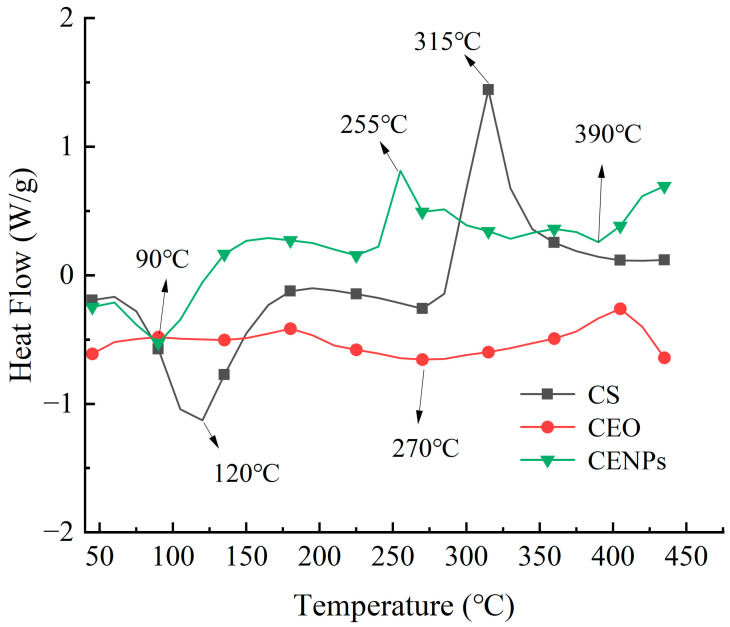
DSC thermograms obtained for samples of CS, CEO, and CENPs.

Differential scanning calorimetry (DSC) is a valuable thermal analysis technique to ascertain the solid complex formation. Abbreviations will be explained when used initially. Figure 2 presents the thermal graphs obtained by DSC for pure CS, pure CEO, and cumin essential oil nanoparticles that incorporate cumin essential oil inside chitosan-embedded nanoparticles (CENPs). The DSC curves reveal a heat absorption peak of CS at approximately 120 °C, indicating its melting and vaporization. As the temperature rises, another exothermic peak occurs at 315 °C, where chitosan experiences pronounced degradation [38]. The broader heat absorption peak at 270 °C is the melting point of CEO, while its decomposition temperature is mentioned. The heat absorption peaks observed in the CENPs at temperatures below 100 °C are due to the evaporation of free water, in contrast to the DSC curves of pure CEO [39]. Furthermore, the nanocapsules’ surface oils displayed minor exothermic peaks because of oxidation at higher temperatures than that of the pure CEO. After the CEO encapsulation, DSC curves demonstrated the shift in peaks, with the CENPs’ heat-absorption peaks increasing from 270 °C to 390 °C. The shift in peak position suggests the interplay between the wall material and the CEOs compounds, resulting in the creation of a novel structural arrangement. Zohuriaan and Shokrolahi’s investigation revealed that the exothermic peaks resulted from the pyrolysis of polysaccharides, leading to the random cleavage of glycosidic bonds and proteins. The exothermic peaks of commonly used food product gums such as chitosan, sodium alginate, and carboxymethylcellulose were found to be close to 300 °C [40]. This information suggests that the nanoencapsulation of cumin essential oil in chitosan can enhance its stability by protecting it from volatilization and oxidation during the heating process.

### 3.3. In Vitro Release of CENPs

**Figure 3:**
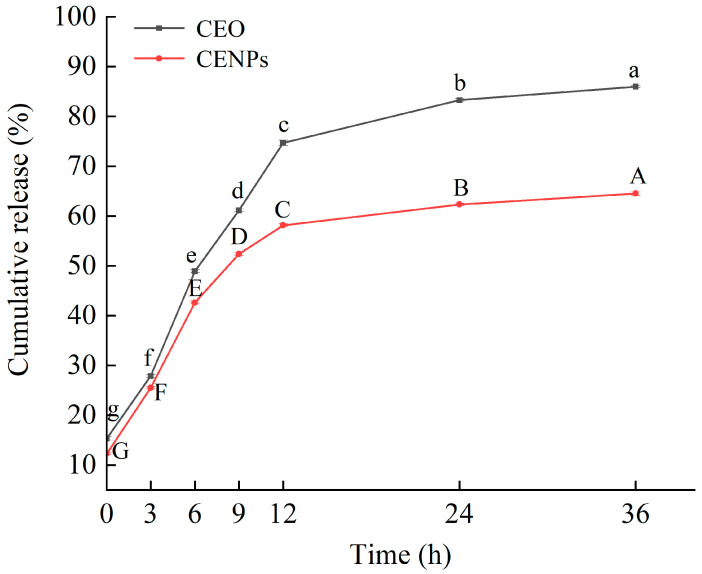
The release effect of CENPs on CEO. Letters “a–g” represent the difference in CEO, and letters “A–G” represent the difference in CENPs. Different letters show statistically significant differences (*p* < 0.05).

Figure 3 exhibits the in vitro profiles of the dissemination of pure CEO and chitosan nano-encapsulated cumin essential oil nanoparticles (CENPs). The quantity of released essential oil was measured at 310 nm during various time intervals [41]. Several mechanisms are in play for the release of essential oils, such as surface erosion, decomposition, diffusion, and desorption from both nanoparticles and microparticles. The in vitro release profiles of pure CEO and CEO from chitosan matrices can be summarized as a two-step biphasic process, with an initial phase of rapid release within the first 12 h. Pure CEO exhibited a release rate of 74.7% after 12 h of storage, whereas CENPs only showed a release rate of 58.2% after 12 h of storage. The burst release observed at the start of the experiment could be attributed to the unembedded CEO situated on the surface of CENPs, alongside the prompt diffusion of embedded CEO neighboring the CENP surface. In the subsequent stage, characterized by a gradual release, the discharge percentage of pure CEO reached 86.0% after 36 h of storage; the release rate of CENPs reached 64.5%. The use of chitosan as a nanocarrier demonstrates its potential for controlling the sustained release of CEO and extending its release time. The swelling of the shell layer of CENPs is responsible for the sustained release behavior of CEO, resulting in the formation of a gel [22,42]. Notably, the gradual release of core compounds occurs through the release of the core material from the coating, the hydrocarbon segment of the coating, and its surface pores [34].

### 3.4. Comparison of Antibacterial Activity between CEO and CENPs

**Figure 4:**
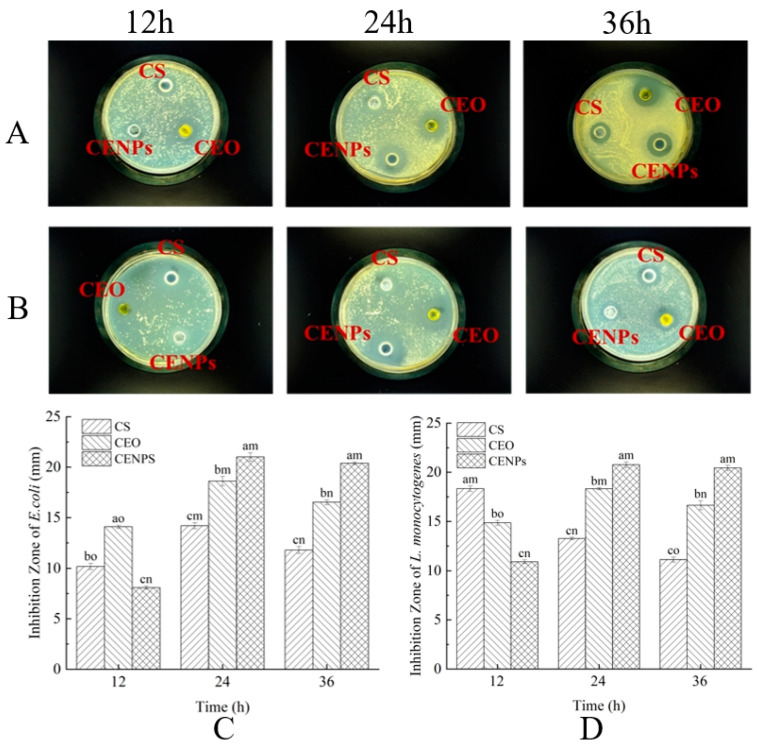
Antibacterial activity of CENPs against *E. coli* (**A**,**C**) and *L. monocytogenes* (**B**,**D**). Letters “a–c” represent the difference between different treatments of the same storage period, and letters “m–o” represent the difference between different storage periods of the same treatment. Different letters show statistically significant differences (*p* < 0.05).

*E. coli* and *L. monocytogenes* constitute prevalent foodborne pathogens. Their numbers, when above a certain threshold, can negatively impact consumer safety, thus making them ideal research subjects in bacteriostatic experiments [43]. In this study, the inhibitory effect of CENPs on *E. coli* and *L. monocytogenes* was investigated, with chitosan and pure CEO serving as control materials in the bacteriostatic tests. As demonstrated in Figure 4A,B, visible inhibition zones of different sizes were evident around the CS, pure CEO, and CENPs on the plate. The antimicrobial potency of chitosan and pure CEO has been established in previous studies. At 12 h, the inhibitory effect of CEO on *E. coli* is evident. The inhibitory circle diameter of *E. coli* was significantly greater when treated with CEO than when treated with CS and CENPs. Monoterpenes found in CEO, including cumin aldehyde, γ-terpinene, limonene, and β-pinene, demonstrated the effective inhibition of *E. coli* propagation [8]. The results of in vitro release tests indicate that the essential oils were rapidly released at this point, resulting in a superior inhibition effect to that of CENPs, which were in a slow-release state (achieved only an 8.09 mm inhibition circle diameter). As the incubation time reached 24 h, the antibacterial activity of CEO and CENPs against *E. coli* increased, resulting in inhibition zones with diameters of 18.61 and 21.01 mm, respectively. However, the inhibitory properties of CS, and CEO against *E. coli* and *L. monocytogenes* began to decrease as the incubation time extended to 36 h. Of the two, the inhibition region of CEO against *E. coli* decreased to 16.53 mm, while that of CENPs was 20.88 mm, with continued strong inhibition capacity. Evidently, the speedy CEO release initially resulted in adequate bacterial inhibition. However, CENPs exhibited enduring bacterial inhibition due to their capacity to gradually release CEO over time.

In contrast to the inhibitory effect on *E. coli*, CS exhibits the strongest inhibition on *L. monocytogenes*, as evidenced by the 18.33 mm diameter of its inhibition zone (Figure 4D) and the results of El-Zehery et al. [44]. This is due to the positive charge of the polysaccharide molecule, which interacts with the negative charge of the microbial membrane through mutual attraction. The protonated NH_3_^+^ groups and negatively charged membrane surface residues are subject to electrostatic attraction, which results in the agglutination of cells and subsequent *L. monocytogenes* cell death [45]. Moreover, the antimicrobial effects of chitosan are influenced by factors such as acetylation and polymerization degree [45]. When the incubation time was increased to 24 h, the antimicrobial effectiveness of CS was lower than that at 12 h, causing a decrease in the diameter of the inhibitory circle to 13.26 mm. This decrease can be attributed to the volatile nature of the acidic chitosan solution and the gradual increase in pH, which led to a reduction in inhibitory effect on microorganisms. The antibacterial efficacy of CEO and CENPs against *L. monocytogenes* at 24 h was superior to that at 12 h. The diameter of the inhibition zone for both increased to 18.33 mm and 20.76 mm, respectively. At 36 h, the diameter of the inhibition zone of CEO decreased to 16.64 mm, whereas the inhibition area of CENPs decreased slightly to 20.44 mm. This suggests that encapsulation had a certain influence on the nanocapsules. The nanocapsules effectively inhibit both *E. coli* and *L. monocytogenes*, and their inhibitory effect was optimized through the slow release of cumin essential oil, thereby controlling the inhibition time.

### 3.5. Microbiological Analyses of Mutton

**Figure 5:**
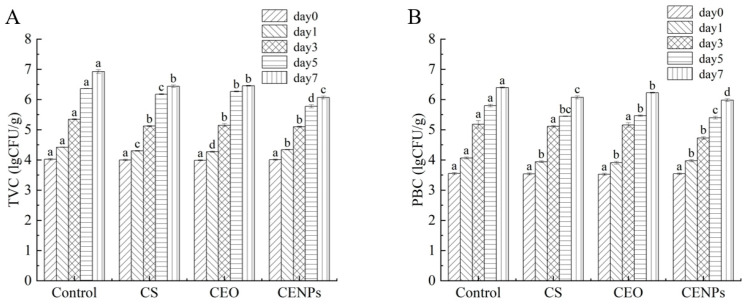
The total viable counts (**A**), and psychrophilic bacteria counts (**B**) changes in the mutton during storage. Different letters show statistically significant differences (*p* < 0.05).

Microbial colonization and metabolism are critical factors in the deterioration of mutton meat during storage. The total viable count (TVC) was utilized as an indicator to assess the level of bacterial contamination and meat product freshness. As illustrated in Figure 5A, the TVC of fresh mutton meat was 4.0 lg CFU/g, whereas the TVC of mutton meat in each treatment group began to increase with an extended storage time. The abundance of nutrients, specifically free amino acids, and water-soluble proteins, produced in frozen mutton meat during storage significantly accelerates the growth of microorganisms in the meat. Similar findings were noted by Pires et al. in their study on chitosan/montmorillonite bio-nanocomposites [46]. After one day of storage, the total viable count (TVC) values of mutton meat increased to 4.42 lg CFU/g (Control), 4.34 lg CFU/g (CS), 4.30 lg CFU/g (CEO), and 4.27 lg CFU/g (CENPs) in the different treatment groups, demonstrating significant differences (*p* < 0.05). By the third day of storage, the TVC values of mutton in the control group were significantly higher than those in the other treatment groups, indicating that cumin essential oil exhibited effective inhibitory effects against bacteria. The antimicrobial activity of CEO was ascribed to the monoterpenes present, including cumin aldehyde, γ-terpene, limonene, and β-pinene, which were found to be the primary compounds of CEO in this particular study [8]. The lipophilic nature of CEO, predominantly due to its monoterpene components, enables the essential oil to disturb the cytoplasmic membrane of microorganisms, ultimately causing cell death [47]. When the mutton meat was stored for 5 days, the total viable count (TVC) values of the blank, CS, and CENP groups exceeded 6.0 lg CFU/g, which is considered the highest acceptable limit for fresh meat according to GB 4789.2-2022. The standard microbiological limit for all meat and meat products is also 6.0 lg CFU/g. Moreover, the TVC values of CS and CENP groups were notably higher than those of the blank group, indicating that chitosan-embedded cumin essential oil nanoparticles were effective in maintaining the edible safety of mutton meat throughout a relatively extended storage period of 5–7 days, through slow release.

Psychrotrophic bacteria are a group of bacteria that can multiply at temperatures below 5 °C and cause spoilage of refrigerated fresh meat [6]. Figure 5B examines the changes in PBC in mutton meat from various treatment groups during cold storage. As shown in the figure, the Control group had significantly more cryophilic bacteria than other samples on the first day of storage (*p* < 0.05) and maintained the highest growth rate throughout the storage period. The mutton meat with added CEO exhibited the least number of psychrotrophic bacteria, attributed to CEO’s inhibitory effect on them. Özogul et al. and Yazgan et al. obtained that nanoemulsion formed by vegetable oil can effectively reduce psychrophilic bacteria counts in fish [48,49]. Mahmoud Hasani-Javanmardi et al. conducted a study on the impact of safflower oil nanoemulsions and cumin essential oil composite oxygen-absorbing packaging on the quality and shelf-life of refrigerated mutton tenderloin [10]. The results showed that the addition of cumin essential oil enhanced the inhibitory effect on psychrotrophic bacteria. The mutton tenderloin was stored for up to three days. The total quantity of cryophilic bacteria observed in the sample group with CEO added significantly exceeded that in the CS and CENP groups. This occurrence could be attributed to the majority of the CEO being volatilized, subsequently resulting in the minimal residual CEO being ineffective in providing the desired inhibitory effect. The CENP group exhibited a significant decrease in the total number of cold-adapted bacteria compared to the CS group. This outcome is attributed to the fusion of the nanoemulsion with the microorganisms’ lipid membrane, which leads to the destabilization of the cytoplasmic membrane and, ultimately, cell death [48,50]. In contrast, CENPs exhibited superior bacterial inhibition compared to CS via CEO release. Through day 7, the overall count of psychrotrophic bacteria in CENPs samples was the least of all at 5.98 lg CFU/g. This indicates that CENPs embedded in chitosan and containing cumin essential oil can effectively hinder the expansion of psychrotrophic bacteria due to gradual cumin essential oil discharge for a specified timeframe.

## 4. Conclusions

Cumin essential oil chitosan nanocapsules (CENPs) were synthesized via an ion–gelation reaction. The resulting CENPs had a uniform spherical structure and were regularly distributed, with a particle size of 584.67 nm and a zeta potential of +33.77 mV. The optimum encapsulation efficiency (EE) of 52% was achieved when the CS/CEO mass ratio was 1:3. Differential scanning calorimetry analysis confirmed that the sodium caseinate had been successfully encapsulated within the CENPs, leading to improved thermal stability. The UV absorption spectroscopy findings indicate that encapsulation actively promotes the sustained release of CEO. Furthermore, the CENPs possess notable antibacterial properties against *E. coli* and *L. monocytogenes*, which was also demonstrated in the application for mutton.

## Figures and Tables

**Table 1:** Average size, zeta potential, PDI, and EE of CENPs.

CS/CEO Ratio	Particle Size (nm)	Zeta Potential (mV)	PDI	EE (%)
1:0.8	810.00 ± 2.70 ^a^	29.03 ± 0.31 ^b^	0.276 ± 0.02 ^b^	38.13 ± 0.35 ^c^
1:1	605.00 ± 1.73 ^c^	25.80 ± 0.32 ^e^	0.292 ± 0.01 ^b^	42.10 ± 0.25 ^b^
1:2	609.33 ± 2.51 ^c^	27.93 ± 0.28 ^c^	0.306 ± 0.02 ^b^	42.04 ± 0.18 ^b^
1:3	584.67 ± 2.62 ^d^	33.77 ± 0.15 ^a^	0.354 ± 0.02 ^a^	52.00 ± 0.27 ^a^
1:4	618.67 ± 2.52 ^b^	27.13 ± 0.21 ^d^	0.143 ± 0.01 ^c^	37.68 ± 0.20 ^c^

Results expressed as mean ± standard deviation (*n* = 3). Different lower-case letters in the same column indicate significant differences (*p* < 0.05).

## Data Availability

The original contributions presented in the study are included in the article, further inquiries can be directed to the corresponding author.
